# The COX-2-Selective Antagonist (NS-398) Inhibits Choroidal Neovascularization and Subretinal Fibrosis

**DOI:** 10.1371/journal.pone.0146808

**Published:** 2016-01-13

**Authors:** Ruoshuang Zhang, Zheli Liu, Han Zhang, Yi Zhang, Dong Lin

**Affiliations:** Department of Ophthalmology, The first affiliated hospital of China Medical University, Shenyang City, liaoning Province, China; Univeristy of Miami, UNITED STATES

## Abstract

Choroidal neovascularization (CNV) is an important pathologic component of neovascular age-related macular degeneration (AMD), and CNV lesions later develop into fibrous scars, which contribute to the loss of central vision. Nowadays, the precise molecular and cellular mechanisms underlying CNV and subretinal fibrosis have yet to be fully elucidated. Cyclooxygenase-2 (COX-2) has previously been implicated in angiogenesis and fibrosis. However, the role of COX-2 in the pathogenesis of CNV and subretinal fibrosis is poorly understood. The present study reveals several important findings concerning the relationship of COX-2 signaling with CNV and subretinal fibrosis. Experimental CNV lesions were attenuated by the administration of NS-398, a COX-2-selective antagonist. NS-398-induced CNV suppression was found to be mediated by the attenuation of macrophage infiltration and down-regulation of VEGF in the retinal pigment epithelium–choroid complex. Additionally, NS-398 attenuated subretinal fibrosis, in an experimental model of subretinal scarring observed in neovascular AMD, by down-regulation of TGF-β_2_ in the retinal pigment epithelium–choroid complex. Moreover, we cultured mouse RPE cells and found that NS-398 decreased the secretion of VEGF and TGF-β_2_ in mouse RPE cells. The results of the present study provide new findings regarding the molecular basis of CNV and subretinal fibrosis, and provide a proof-of-concept approach for the efficacy of COX-2 inhibition in treating subretinal fibrosis.

## Introduction

Choroidal neovascularization (CNV) is an important pathologic component of neovascular age-related macular degeneration (AMD), which may lead to severe loss of central vision in elderly individuals [1]. The pathogenesis of CNV is known to be multifactorial, involving patient age, metabolic dysfunction, sun damage, oxidative stress, circulatory disturbances, and inflammatory immune response [2–4]. In recent studies, the inflammatory immune response was shown to be the key factor in the formation and progression of CNV, which required continuous and complex interactions among inflammatory factors, cytokines and the extracellular matrix [4–5]. VEGF is a cytokine that plays a decisive role in angiogenesis and CNV formation [6–8]. Of late, VEGF inhibition has become the main approach for the clinical treatment of CNV [9–12]. However, even with standardized and repeated anti-VEGF treatment, only 30%–40% of exudative AMD patients demonstrate vision improvement, and approximately 8% of exudative AMD patients eventually experience a loss in visual acuity that leads to blindness after treatment; some patients experience expansion from subretinal fibrosis, resulting in vision loss [13–16]. In view of the complexity of CNV formation, the effect of anti-VEGF treatment is limited. Therefore, we should find new therapeutic targets and explore a more reasonable and effective method for CNV treatment.

Cyclooxygenase (COX) is a bifunctional rate-limiting enzyme involved in the inflammatory immune response. Three isoforms of COX have been identified, COX-1, COX-2, and COX-3 [17–21]. COX-1 is constitutively expressed in almost all tissues and is believed to be responsible for maintaining levels of prostaglandins for various housekeeping functions [18]. COX-2 is induced by pathologic stimuli, including cytokines, growth factors, inflammatory mediators, and bacterial lipopolysaccharides [20]. COX-3 is encoded by the same gene as COX-1, but it is not functional in humans [21]. COX-2 has been confirmed to play an important role in inflammatory reactions. CNV is regarded to be the result of a chronic inflammatory process involving macrophage infiltration and interactions among inflammatory factors, cytokines and the extracellular matrix [4–5]. Some reports have shown that COX-2 was present in RPE cells and COX-2 null mice exhibited significantly less CNV formation associated with reduced expression of VEGF [22–24]. However, the relationship between COX-2 and CNV is not fully understood. Subretinal fibrosis is closely correlated with the upregulation of TGF-β, especially TGF-β_2_, in CNV associated with AMD [25–26]. A recent study showed that COX-2 could stimulate macrophages to produce TGF-β, which consequently synthesize and deposit collagen fibers, eventually leading to fibrosis [27]. COX-2 is present in RPE cells, and the relationship between COX-2 and subretinal fibrosis is unclear.

In the present study, we found that a COX-2-selective inhibitor (NS-398) can attenuate CNV and subretinal fibrosis lesions by suppressing macrophage infiltration and downregulating VEGF and TGF-β_2_, respectively. Thus, we demonstrate a proof-of-concept approach for the utility of COX-2 inhibition in investigating the molecular basis of CNV and the treatment of subretinal fibrosis.

## Materials and Methods

### 2.1. Animals

C57BL/6 mice that were 7–10 weeks old were obtained from China Medical University. All mice were treated according to the Association for Research in Vision and Ophthalmology (ARVO) Statement for the Use of Animals in Ophthalmic and Vision Research. The Institutional Animal Care and Use Committee of China Medical University approved this research.

### 2.2. Induction and assessment of CNV and subretinal fibrosis in mice

The C57BL/6 mice were anesthetized, and their pupils were dilated. As described in detail previously, laser photocoagulation was applied around the optic disc using a 532-nm diode laser (0.1s, 200 mW, 75μm, Iridex, Mountain View, CA, USA) to burn the posterior pole of the retina (4 spots/eye) [28]. Only lesions in which a subretinal bubble developed were used in subsequent experiments in the absence of subretinal hemorrhage. Ten days after photocoagulation, the mice were perfused with 50 mg/mL of fluorescein-labelled dextran (MV2×10^6^, Sigma, USA) in the left ventricle. The eyes were enucleated and fixed in 4% paraformaldehyde (Boster, Wuhan, China). Then the anterior segments and lenses were removed to create eye cups. Each eye cup was radially cut into four quadrants to make choroidal flat mounts. The total area of hyperfluorescence associated with each burn was measured using the ImageJ software (National Institutes of Health, Bethesda, MD).

To establish a subretinal fibrosis model, the C57BL/6 mice were intraperitoneally injected with 2.5 mL of sodium thioglycolate (Sigma, USA). After 3 days, peritoneal exudate cells (PECs) were extracted from the abdominal cavity. As described in detail previously, after laser photocoagulation (0.1s, 200 mW, 75μm, 532 nm diode laser, Iridex, Mountain View, CA, USA), PECs were injected under laser photocoagulation spots using a blunt-tipped needle. Only lesions that exhibited focal retinal detachment were used in subsequent experiments. This method has been confirmed to successfully induce subretinal fibrosis in a mouse model and has been applied in many important studies [28–29]. A previous study has reported the utility of glial fibrillary acidic protein (GFAP) staining in effectively assessing subretinal fibrosis in an animal model [28]. According to this method, on day 7, polyclonal rabbit anti-GFAP antibody (1:400, Dako, Glostrup, Denmark) and FITC-conjugated anti-rabbit IgG (Invitrogen) were used to stain and detect GFAP in PEC-injected choroidal flat mounts. GFAP staining was observed using a fluorescence microscope and was quantified using ImageJ.

### 2.3. Cultures of RPE cells from mice

The eyes were removed from the C57BL/6 mice (n = 4). Following enucleation, the corneas and lenses were removed to form eyecups. The eyecups were digested in 0.2% trypsin. Then, the RPE cells were dissected and grown in DMEM/CM media containing DMEM, 20% FBS, 1% penicillin–streptomycin, 1% non-essential amino acids, and 1%L-glutamine. Cytokeratin 8 is an intermediate filament protein specifically expressed in epithelial and RPE cell [30]. In an immunostaining study, all cells were cytokeratin-8 positive and were used in this study.

### 2.4. NS-398 treatment in an animal model of CNV and RPE culture

The mice were intraperitoneally treated with a COX-2-selective antagonist (NS-398) (30mg/kg, Sigma, USA) or phosphate-buffered saline (PBS, used as a vehicle) or MF1 (25mg/kg, Sigma, USA) + DC101 (25mg/kg, Sigma, USA) 1 h before photocoagulation, and treatments were continued daily until the end of the study. MF1 and DC101 are neutralizing antibodies specific for mouse VEGFR1 (MF1) and VEGFR2 (DC101) as a positive control [31].

Mouse RPE cultures were washed with serum-free media (SFM), incubated in SFM for 12 h, washed again with SFM, and further incubated for 12 h in six-well plates. NS-398 (0 ng/mL, 25 ng/mL, 50 ng/mL, and 100 ng/mL) was added to the cultures. After 1 h, TNF-α (10 ng/mL; Sigma, USA) was added to these plates. Some studies showed that TNF-α could stimulate RPE cells to effectively secrete many cytokines, such as VEGF and TGF-β_2_ in vitro [32].

### 2.5. Immunofluorescence

In the CNV model, the eyes were enucleated on the third day after photocoagulation. As described previously, we used the FITC-conjugated endothelial cell marker isolectin B4 (1:200, Vector Laboratories) and the RPE-conjugated rat anti-mouse macrophage marker F4/80 (1:100, Invitrogen, Carlsbad, CA) for double immunofluorescence staining of the choroidal flat mounts [28]. ImageJ was used to measure the total area covered by the F4/80-positive cells within and adjacent to laser-induced lesions and normalized to total area of CNV (defined as 100%).

### 2.6. Measurements of VEGF and TGF-β_2_ with qRT-PCR

In the CNV and the subretinal fibrosis models, RPE–choroid complexes were isolated on days 1, 3, 5, and 7 after model establishment. We used TRIzol reagent (TaKaRa, Japan) to extract total RNA from the RPE–choroid complexes. Subsequently, the mRNA was reverse transcribed using a PrimeScript RT reagent Kit (TaKaRa, Japan), according to the manufacturer’s instructions. qRT-PCR was performed using SYBR green real-time PCR mix (TaKaRa, Japan).

In the RPE cultures, total RNA was extracted using the TRIzol reagent. Then, we used the same steps to perform qRT-PCR. Target sequences were amplified using the following primer pairs: VEGF, 5′-GTTCACTGTGAGCCTTGTTCAG-3′ and 5′-GTCACATCTGCAAGTACGTTCG-3′; TGF-β2, 5′-GGATGGAAATGGATCCATGAACCC-3′ and 5′-TGTTGTACAGGCTGAGGACTTTGG-3′; and β-actin, 5′-GATGACCCACAGATCATGTTTGA-3′ and 5′-GGAGAGCATAGCCCTCGTAG-3′. All estimated mRNA levels were normalized to β-actin mRNA levels.

### 2.7. Enzyme-linked immunosorbent assay

The RPE–choroid complexes were isolated on days 1, 3, 5, and 7 after CNV and subretinal fibrosis formation, and they were immersed in tissue protein extraction reagent (T-PER; IL) supplemented with a protease inhibitor cocktail (Halt, Pierce). Cell culture supernatants were removed and diluted. Then, the soluble protein levels of VEGF and TGF-β_2_ in the lysate and supernates were tested by the corresponding mouse VEGF and TGF-β_2_ kits (R&D Systems, MN), according to the manufacturer’s instructions.

### 2.8. Statistical Analysis

All data are presented as mean ± standard deviation (SD). Each result is representative of at least three independent experiments. Comparisons of mean values were performed using Student’s t-test (SPSS, Chicago, IL). P-values <0.05 were considered to statistically significant.

## Results

### 3.1. Suppression of CNV and VEGF with NS-398

**Fig 1:**
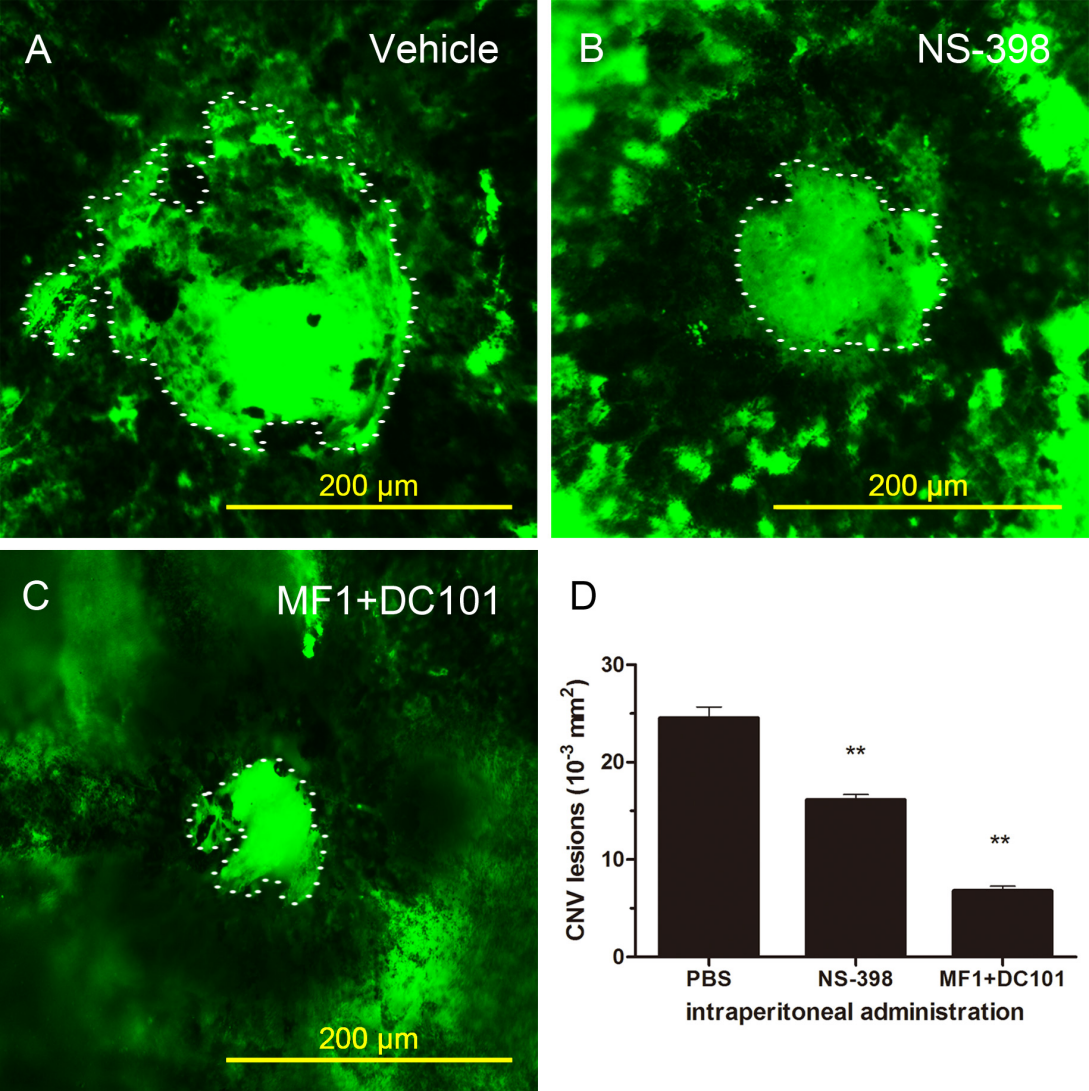
Suppression of CNV with COX-2-selective Antagonist (NS-398). Representative images of fluorescein dextran perfused RPE–choroidal flat mounts of mice administered PBS vehicle (A), NS-398 intraperitoneally (B), MF1+ DC101 (C) day 10 after photocoagulation. The CNV lesions were assessed quantitatively (D). **P<0.001 compared with vehicle-treated mice. Error bars represented ±SD. intraperitoneal vehicle, n = 18 burn spots, intraperitoneal NS-398, n = 20 burn spots, intraperitoneal MF1+ DC101, n = 16 burn spots, Scale bars in A, B,C are 200μm.

The typical features of experimental CNV in the PBS-treated mice after photocoagulation on day 10 are shown in Fig 1A. In contrast, we observed a few new vessels in the mice treated with NS-398 or MF1+DC101, respectively (Fig 1B and 1C). An assessment of CNV lesions on the choroidal flat mounts showed that NS-398 or MF1+DC101 significantly inhibited the CNV areas respectively on day 10 (**P<0.001, Fig 1D).

### 3.2. Suppression of macrophage infiltration in NS-398-treated eyes

**Fig 2:**
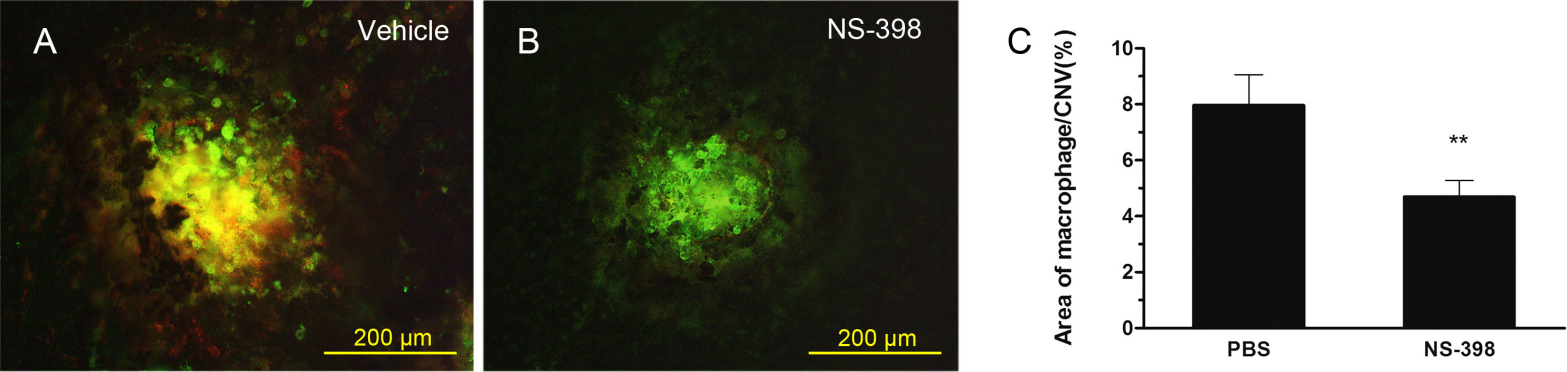
Suppression of macrophage infiltration with COX-2-selective Antagonist (NS-398). Green fluorescence from isolectin B4 indicates CNV, red fluorescence indicates F4/80-positive macrophages. Representative macrophage infiltration lesions in the PBS vehicle-treated mice (A) and NS-398-treated mice (B). Areas of F4/80-positive cells were assessed quantitatively (C). **P<0.001 compared with vehicle-treated mice. Error bars represented ±SD. intraperitoneal vehicle, n = 15 burn spots, intraperitoneal NS-398, n = 16 burn spots, Scale bars in A, B are 200μm.

A macrophage-specific marker (F4/80) was used to assess macrophage infiltration in CNV lesions on choroidal flat mounts. The F4/80-positive cells were centralized within and around laser spots 3 days after photocoagulation (Fig 2A). Macrophage infiltration was significantly attenuated, and the number of F4/80-positive cells was decreased in the NS-398-treated mice (Fig 2B). The quantitative assessment of the macrophage infiltration areas demonstrated a 31.7% reduction in F4/80 positivity in the NS-398-treated mice 3 days after photocoagulation (**P < 0.001 Fig 2C).

### 3.3. Suppression of subretinal fibrosis with NS-398

**Fig 3:**
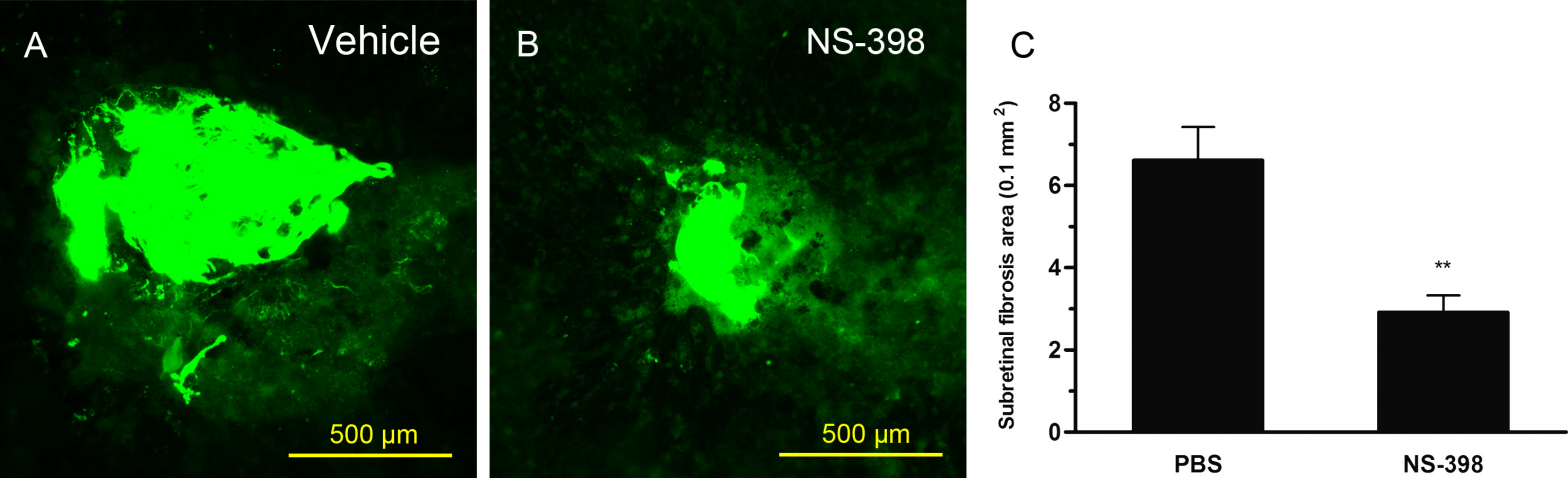
Suppression of subretinal fibrosis with COX-2-selective Antagonist (NS-398) in a mouse model. Representative subretinal fibrosis of RPE–choroidal flat mounts of mice administered PBS vehicle (A), NS-398 intraperitoneally (B), on day 7 after PECs-injecting. Subretinal fibrosis lesions were assessed quantitatively(C), **P<0.001 compared with vehicle-treated mice. Error bars represented ±SD. intraperitoneal vehicle, n = 16 burn spots, intraperitoneal NS-398, n = 18 burn spots, Scale bars in A, B are 500μm.

The subretinal fibrosis model was established by injecting PECs into the subretinal space, which was similar to fibrotic subretinal scarring caused by neovascular AMD [29]. This animal model showed typical features of experimental subretinal fibrosis in the PBS-treated mice on day 7 after PEC injection (Fig 3A). In contrast, subretinal fibrosis was attenuated in the mice treated with NS-398 (Fig 3B). A quantitative assessment of subretinal fibrosis lesions indicated that NS-398 significantly reduced the subretinal fibrosis areas by 33% (*P* < 0.001) (Fig 3C). These results suggest that COX-2 signaling is involved in the pathogenesis of subretinal fibrosis.

### 3.4. Suppression of VEGF and TGF-β2 with NS-398 in CNV and subretinal fibrosis models and mouse RPE cells

**Fig 4:**
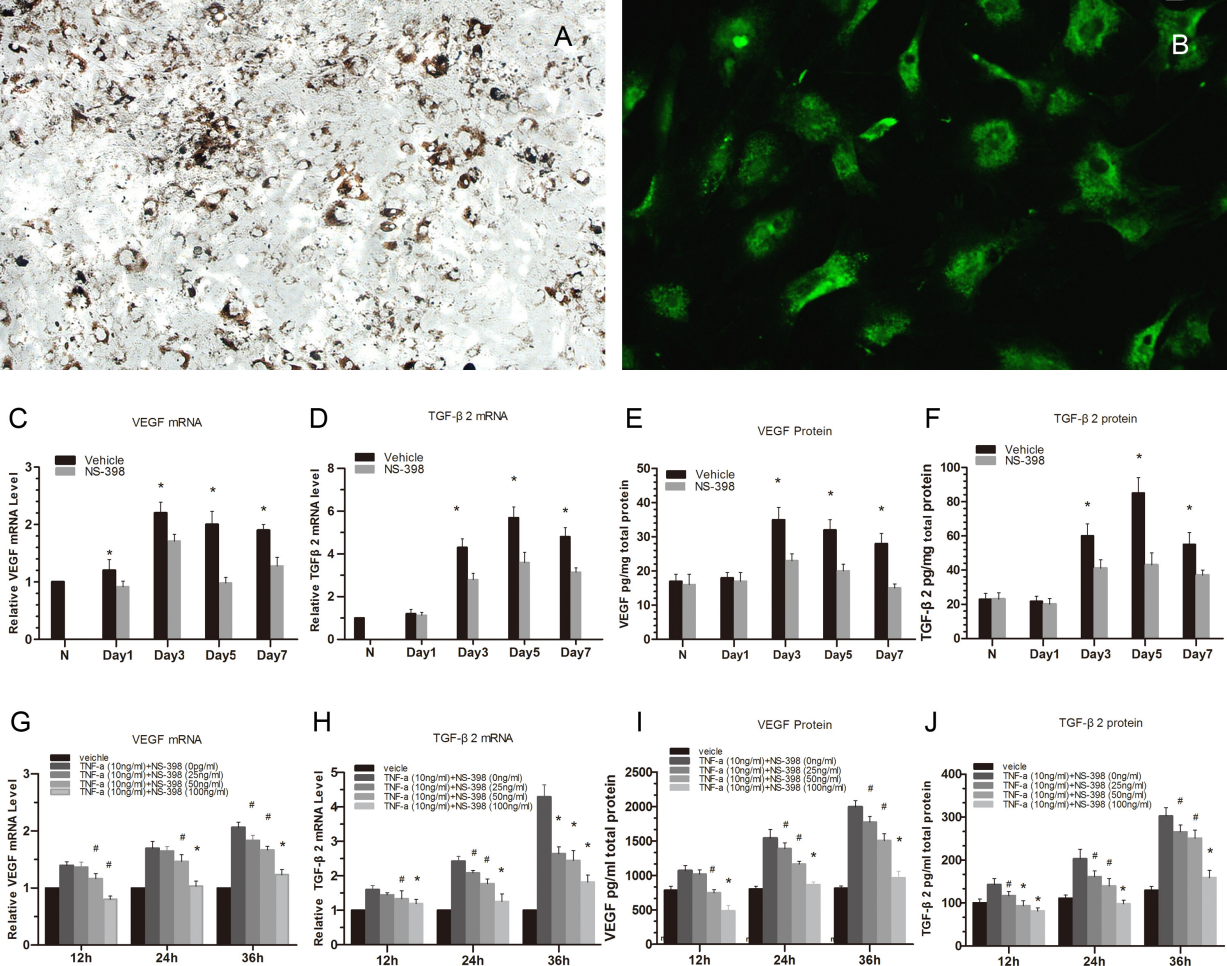
Suppression of VEGF and TGF-β2 expression with COX-2-selective Antagonist (NS-398) in RPE–choroid complexes and mouse RPE cells. Light microscope images of confluent RPE cultures on day 10 (A) (magnification = 100×). Immunofluorescence showed Cytokeratin 8-positive cells were the RPE cells (B) (magnification = 400×). In the RPE–choroid complexes, the mRNA and protein levels of VEGF and TGF-β_2_ were suppressed significantly in the NS-398-treated mice (4C-4F). *P<0.01 compared with vehicle-treated mice for all, after CNV and subretinal fibrosis model formation; vehicle n = 6 eyes for each time point, NS-398 n = 6 eyesfor each time point. In mouse RPE cells, the mRNA and protein levels of VEGF (G, I), TGF-β_2_ (H, J) were significantly reduced respectively at 12h, 24h, 36h after added NS-398 (4G-4J). (^#^*P*<0.05, *P<0.01). Error bars indicate mean ± SD; Experiments were conducted in triplicate with similar results.

VEGF is a cytokine that plays a decisive role in CNV formation [6–7]. TGF-β_2_ is closely correlated with subretinal fibrosis in CNV associated with AMD [25–26]. To elucidate the molecular mechanisms involved in the regulation of CNV and subretinal fibrosis by NS-398-induced COX-2 inhibition, we found that the mRNA and protein levels of VEGF in CNV model (*P* < 0.01) (Fig 4C and 4E) and TGF-β_2_ in subretinal fibrosis model (*P* < 0.01) (Fig 4D and 4F) were significantly reduced in the NS-398-treated mice compared with those of the PBS-treated mice on days 1, 3, 5, and 7 after model establishment. These results revealed the molecular mechanisms of CNV and subretinal fibrosis development.

The RPE cells became confluent on day 10 (Fig 4A). The hexagonal cells, which were cytokeratin-8 positive with green fluorescence, were proved to be RPE cells (Fig 4B). We successfully stimulated the RPE cells to effectively secrete many cytokines, such as VEGF and TGF-β_2_, using TNF-α (10 ng/mL) [32]. Using this cell model, the mRNA and protein levels of VEGF (Fig 4G and 4I) and TGF-β_2_ (Fig 4H and 4J) were significantly elevated in the TNF-α-treated RPE cells. In contrast, the mRNA and protein levels of VEGF (*P<0.01) (Fig 4G and 4I) and TGF-β2 (*P<0.01) (Fig 4H and 4J) were reduced at 12, 24, and 48 h after the addition of NS-398 (25 ng/mL, 50 ng/mL, and 100 ng/mL respectively). These results revealed that COX-2 signaling was involved in cytokine production.

## Discussion and Conclusion

The results of our study demonstrate the relationship between COX-2 signaling and CNV and subretinal fibrosis. COX-2 is expressed in a laser-induced CNV model [33]. Herein, we report attenuation of CNV with the administration of NS-398 via an inhibitory effect on macrophage accumulation and a reduction in the expression of proangiogenic factors. To the best of our knowledge, the present study is the first to characterize the relationship between COX-2 and subretinal fibrosis. We found that NS-398 attenuated the formation and development of experimental subretinal fibrosis similar to subretinal scarring in neovascular AMD. Moreover, we cultured mouse RPE cells and used TNF-α to make an experimental in vitro model. We found that NS-398 decreased the secretion of VEGF and TGF-β_2_ in mouse RPE cells. Furthermore, findings from the vitro experiment explained the molecular mechanisms of NS-398-induced CNV and subretinal fibrosis suppression.

COX is a bifunctional rate-limiting enzyme involved in the inflammatory immune response. COX-2 is rapidly induced in response to a number of pathophysiological conditions [34]. Under pathophysiological conditions, COX-2 is constitutively expressed in a number of cell and tissue types including the nasal mucosa [35], bronchial epithelial cells [36], medullary cells of the kidney [37], choroid plexus of the developing brain [38], smooth muscle cells [39], and vascular endothelial cells [40]. In the eyes, COX-2 has been detected in RPE cells stimulated by inflammatory cytokines [41]. Previous studies have indicated that COX-2 overexpression is involved in angiogenesis by producing angiogenic cytokines, which induces the migration of endothelial cells and formation of vascular tube [42]. In the present study, we created a laser-induced CNV mouse model and assessed the utility of NS-398. Laser photocoagulation has been demonstrated as a method of inducing CNV in animal models. Bruch’s membrane is ruptured by high laser energy, with various proangiogenic and proinflammatory factors produced in response, leading to the development of CNV. As described previously, although the pathogenesis of the CNV model is different from that of AMD, CNV formation is believed to involve similar mechanisms, with the RPE and endothelial cells shown to secrete identical proangiogenic factors during the development of CNV [28]. NS-398, N-[2-cyclohexyloxy-4-nitrophenyl] methanesulfonamide, is a potent and specific COX-2-selective inhibitor with an affinity for COX-2 binding sites that it similar to that of COX-2 itself [43–45]. Previous studies have revealed that NS-398 significantly suppresses tumor development by reducing VEGF and tumor-related angiogenesis in an experimental tumor model [46]. Our research shows that the areas of laser-induced CNV were reduced in the mouse model intraperitoneally injected with NS-398 without retinal destruction. We posit the utility of NS-398 in investigating the molecular basis of CNV.

A study has demonstrated macrophage accumulation and VEGF upregulation during CNV pathogenesis [47]. Macrophage depletion diminishes lesion size and reduces VEGF in an experimental mouse CNV model [48]. In prostate cancer cells, NS-398 attenuates macrophage accumulation by increasing macrophage migration inhibitory factor expression [49]. In our present study, NS-398 reduced macrophage accumulation and infiltration while simultaneously diminishing CNV lesion size. These data further support the hypothesis that the pathological process of CNV is associated with chronic inflammation and suggests that anti-inflammatory therapy, such as a COX-2-selective inhibitor, should be effective for the treatment of CNV. In addition to intraperitoneal injection, further studies are necessary to investigate the penetration, intraocular pharmacokinetics, toxicology and effects on retinal ultrastructure and function from the intravitreal administration of NS-398.

It is recognized that as a result of the wound healing process, CNV lesions later develop into fibrous scars, which contribute to the loss of central vision. A recent study documented the development of subretinal fibrosis after anti-VEGF treatment in neovascular AMD [50]. Subretinal fibrosis was shown to be related to an imbalance between angiogenesis and tissue fibrosis after anti-VEGF therapy [50]. Therefore, we should find new therapeutic targets and explore a more reasonable and effective method for CNV and subretinal fibrosis treatment. COX-2 may be a potential therapeutic target that affects various fibrotic processes. For example, COX-2 inhibitors reduce the secretory activity of macrophages, eventually leading to the inhibition of fibrosis in a chronic pancreatitis animal model after NS-398 was added [27]. These findings are consistent with our results where NS-398 induced subretinal fibrosis attenuation in an animal model.

In the eyes, research has recorded the relationship between TGF-β and various ocular inflammatory, proliferative, and degenerative diseases [26, 51]. TGF-β has three mammalian isoforms, TGF-β_1_, TGF-β_2_ and TGF-β_3_, each encoded by different genes and located on different chromosomes [52–53]. It has been confirmed that TGF-β_2_ is the predominant isoform in the neural retina and RPE–choroid complexes [54]. We report that VEGF and TGF-β_2_ were upregulated in vivo and in vitro. NS-398 significantly attenuated CNV-related VEGF and subretinal fibrosis-related TGF-β_2_.

In our previous study, we characterized the relationship between Platelet-activating factor receptor (PAF-R) signaling and CNV [55]. We observed that CNV was suppressed by blocking PAF-R using the novel receptor antagonist WEB2086 and WEB2086-dependent suppression of CNV occurred via the inhibition of macrophage infiltration and the expression of proangiogenic (VEGF) and proinflammatory molecules (monocyte chemotactic protein-1 and IL-6) in the RPE–choroid complex. We also reported that WEB2086-induced PAF-R blockage suppressed experimentally induced subretinal fibrosis in the study. We utilize the similar protocol to demonstrate the role of COX-2 in experimental CNV and subretinal fibrosis. Some studies suggested that PAF could up-regulate the expression of COX-2 in different cell cultures, including peritoneal cells, corneal epithelium, esophageal cancer cells and alveolar macrophages [56–58]. We postulate that PAF may also involve in COX-2 expression in RPE cells. Further studies are necessary to evaluate the relationship between PAF and COX-2 in CNV development.

In summary, the present study is the first to demonstrate that a COX-2-selective inhibitor reduces subretinal fibrosis in vivo and in vitro. We report the cellular and molecular mechanisms of NS-398-induced CNV attenuation, including inhibitory effects, on macrophage accumulation and decreased proangiogenic expression. The results of the present study demonstrate a proof-of-concept approach for the use of NS-398 in investigating the molecular basis of CNV and treating subretinal fibrosis.

## Supporting Information

S1 Fig Light microscope confirmed that the structure of retina is normal. Therefore, we concluded that no retinal toxicity from the intravitreal administration of NS-398 at this dose.
(TIF)
