# Supplementary figures and images for: The COX-2-Selective Antagonist (NS-398) Inhibits Choroidal Neovascularization and Subretinal Fibrosis
Source: PLoS One. 2016 Jan 13;11(1):e0146808. doi: 10.1371/journal.pone.0146808 (PMC4711821; doi:10.1371/journal.pone.0146808)

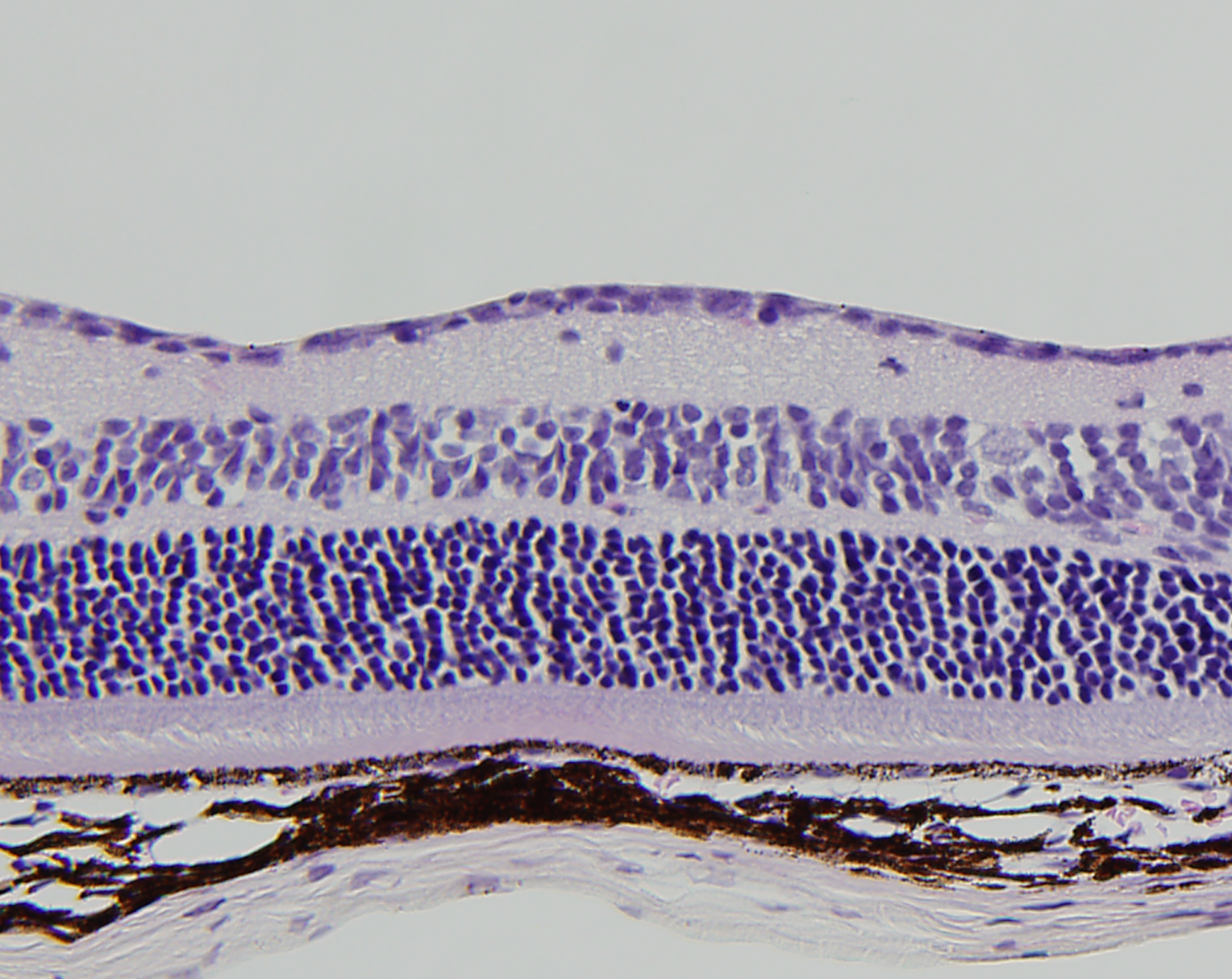

Supplement: S1 Fig — Therefore, we concluded that no retinal toxicity from the intravitreal administration of NS-398 at this dose. (TIF) [file pone.0146808.s001.tif]
